# Letter from the Editor in Chief

**DOI:** 10.19102/icrm.2020.110607

**Published:** 2020-06-15

**Authors:** Moussa Mansour


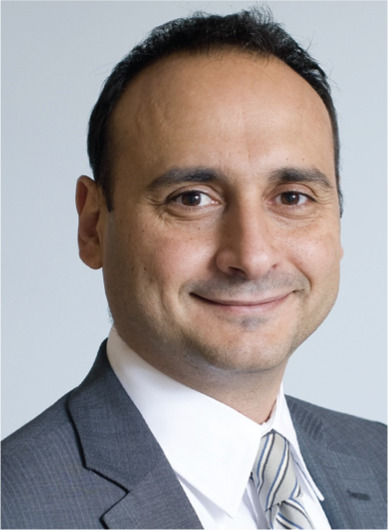


Dear Readers,

Among the landmark clinical trials presented last month during the HRS 2020 Science online broadcast was the Review of the Safety and Effectiveness of the THERMOCOOL SMARTTOUCH^®^ SF Catheter Evaluated for Treating Symptomatic Persistent Atrial Fibrillation (PRECEPT) trial.^1^ In this prospective, multicenter study, 348 patients across 27 centers in the United States and Canada underwent pulmonary vein (PV) isolation (PVI) alone (55.5%) or PVI plus additional ablation targets (mainly the posterior left atrial wall and non-PV triggers induced by isoproterenol) (44.5%) in a stepwise approach. Patients were followed for 15 months with stringent arrhythmia monitoring. The primary effectiveness endpoint, which included freedom from atrial arrhythmias lasting longer than 30 seconds, was achieved in 62% of patients. The primary safety endpoint was also met, with a remarkably low rate of adverse events (4.1%). Further, clinical success was achieved in 80% of patients, 86.1% of patients remained free from repeat ablation, and there was a significant reduction in health-care resource utilization.

An indirect comparison of the workflows and atrial arrhythmia recurrence rates between PRECEPT and other multicenter persistent atrial fibrillation (AF) studies such as STAR AF II,^2^ STOP Persistent AF,^3^ TOUCH AF,^4^ and CRYO4PERSISTENT AF^5^ suggests better outcomes exist in PRECEPT. The PRECEPT trial is also significant as the first prospective multicenter study performed using contact force sensing. It demonstrated that freedom from atrial arrhythmias can be achieved in a large percentage of patients, while suggesting that, in persistent AF, the ablation of non-PV targets in addition to PVI is necessary. Future work involving the mapping of non-PV targets is of the utmost importance.

Persistent AF ablation is likely to proliferate as paroxysmal AF ablation did more than one decade ago. Future research should emphasize patient-centric outcomes such as AF burden reduction and long-term health-care use to guide treatment decisions in this area.

Sincerely,


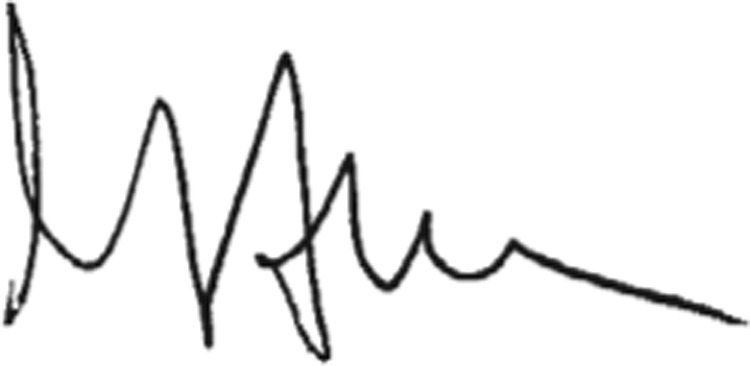


Moussa Mansour, md, fhrs, facc

Editor in Chief

The Journal of Innovations in Cardiac Rhythm Management

MMansour@InnovationsInCRM.com

Director, Atrial Fibrillation Program

Jeremy Ruskin and Dan Starks Endowed Chair in Cardiology

Massachusetts General Hospital

Boston, MA 02114
